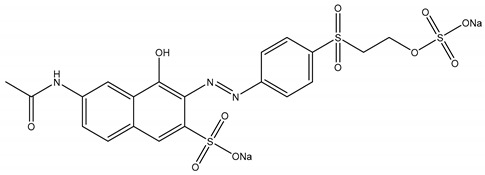# Phosphoric Acid Activated Carbon from *Melia azedarach* Waste Sawdust for Adsorptive Removal of Reactive Orange 16: Equilibrium Modelling and Thermodynamic Analysis

**DOI:** 10.3390/molecules25092118

**Published:** 2020-05-01

**Authors:** Jehanzeb Ali Shah, Tayyab Ashfaq Butt, Cyrus Raza Mirza, Ahson Jabbar Shaikh, Muhammad Saqib Khan, Muhammad Arshad, Nadia Riaz, Hajira Haroon, Syed Mubashar Hussain Gardazi, Khurram Yaqoob, Muhammad Bilal

**Affiliations:** 1 Department of Environmental Sciences, COMSATS University Islamabad, Abbottabad Campus, Abbottabad, KPK 22060, Pakistan; jehanzeb360@gmail.com (J.A.S.); saqi78@gmail.com (M.S.K.); nadiariazz@cuiatd.edu.pk (N.R.); hajira@uoh.edu.pk (H.H.); mbshrgardazi945@gmail.com (S.M.H.G.); 2 Department of Civil Engineering, University of Hail, Hail, Hail Province 55476, Saudi Arabia; ta.butt@uoh.edu.sa (T.A.B.); crazamirza@gmail.com (C.R.M.); 3 Department of Chemistry, COMSATS University Islamabad, Abbottabad Campus, Abbottabad, KPK 22060, Pakistan; ahson@cuiatd.edu.pk; 4 Department of Environmental Science, IESE, National University of Science and Technology, Islamabad 44000, Pakistan; marshad@iese.nust.edu.pk; 5 Department of Environmental Sciences, University of Haripur, Haripur, KPK 22620, Pakistan; 6 Department of Botany, Women University of Azad Jammu and Kashmir, Bagh, Azad Kashmir 12500, Pakistan; 7 School of Chemical and Materials Engineering, National University of Science and Technology, Islamabad 44000, Pakistan; khurram.yaqoob@scme.nust.edu.pk

**Keywords:** isotherm and kinetics modelling, activated carbon, waste sawdust, error analysis, ortho-phosphoric acid, reactive orange 16

## Abstract

Waste wood biomass as precursor for manufacturing activated carbon (AC) can provide a solution to ever increasing global water quality concerns. In our current work, *Melia azedarach* derived phosphoric acid-treated AC (MA-AC400) was manufactured at a laboratory scale. This novel MA-AC400 was tested for RO16 dye removal performance as a function of contact time, adsorbent dosage, pH, temperature and initial dye concentration in a batch scale arrangement. MA-AC400 was characterized via scanning electron microscopy, energy dispersive X-ray spectroscopy, Fourier transform infrared spectroscopy, dynamic light scattering (DLS) and fluorescence spectroscopy. MA-AC400 is characterized as mesoporous with BET surface area of 293.13 m^2^ g^−1^ and average pore width of 20.33 Å. pH_PZC_ and Boehm titration confirm the acidic surface charges with dominance of phenolic functional groups. The average DLS particle size of MA-AC400 was found in the narrow range of 0.12 to 0.30 µm and this polydispersity was confirmed with multiple excitation fluorescence wavelengths. MA-AC400 showed equilibrium adsorption efficiency of 97.8% for RO16 dye at its initial concentration of 30 mg L^−1^ and adsorbent dose of 1 g L^−1^. Thermodynamic study endorsed the spontaneous, favorable, irreversible and exothermic process for RO16 adsorption onto MA-AC400. Equilibrium adsorption data was better explained by Langmuir with high goodness of fit (R^2^, 0.9964) and this fitness was endorsed with lower error functions. The kinetics data was found well fitted to pseudo-second order (PSO), and intra-particle diffusion kinetic models. Increasing diffusion constant values confirm the intraparticle diffusion at higher RO16 initial concentration and reverse was true for PSO chemisorption kinetics. MA-AC400 exhibited low desorption with studied eluents and its cost was calculated to be $8.36/kg.

## 1. Introduction

Synthetic colorants are widely applied chemicals in the textile sector. A diverse variety of these complex dyes is available on the international market with a range of properties and physiognomies. A significant amount of these toxic dyes is directly dumped every year into the aquatic bionetwork directly in the form of industrial effluents. The multifaceted aromatic chemistry of such complex dyes offers resistance to biodegradation, resulting in incomplete mineralization, thus metabolites of varying toxicity are produced [1] which therefore increase the environmental health concerns. Presently, around 10,000 types and 700,000 tons of different dyes and pigments are manufactured worldwide annually with ever increasing trend [2]. The reactive dyes class represents a major share of commercial grade synthetic colorants due to their excellent capability of forming covalent bonding between the reactive groups of the dyes and the functional groups of substrates like cellulose in the case of textile products [3]. Dye-bath facilities lose a significant portion of these dyes due to hydrolysis reactions. The constituents of these organic chemicals are enormously noxious, consequentially leading towards skin and skin sensitivity, mutagenicity, carcinogenicity and eye irritation, etc. [4,5,6]. Their threat level increases with their stable chemical nature due to aromatic rings and other intermediates produced on fragmentation. These health issues necessitate the treatment of such hazardous dyes in effluents prior to their dumping into the environment.

Currently, conventional technologies being adopted for the treatment of reactive dyes include photocatalytic degradation [7], enzymatic action [8], chemical coagulation/flocculation [9], ion exchange [10] and membrane separation [11], etc. At an industrial scale these conventional treatment processes have certain limitations: e.g., phase, shape, band gap, light source, structure, reactor design and catalyst recovery [12], the temperature specificity and alkaline conditions of laccase enzyme [13], residual sludge generation during chemical coagulation [14], slow pore diffusion, low accessible flow rates, high pressure drop and flow channeling in ion exchange [15], and chemical incompatibility in membrane separation [16], etc. Therefore, it is a pressing need for a treatment technology to solve the problem in a sustainable and cost-effective manner.

Adsorption appears to be an economical, effective and eco-friendly technology for the elimination of reactive dyes from effluents when compared with other methods due to its numerous advantages, which include low cost, ease of design at a large scale, facile manipulations, resistance to contaminants, higher efficiency rate, and an aptitude to cope with dilute as well as concentrated dye effluents [17]. Generally, a wide range of adsorbents obtained from variety of sources such as clay [18], nanoparticles [19], nanotubes [20], agricultural- and forest-derived wastes [21,22], activated carbon [23], graphene composites [24], zeolites [25], chitosan [26], and a number of polymeric adsorbents [27] have been employed for the removal of reactive dyes. The high surface area, good adsorption capacity, mechanical and chemical stability of activated carbon (AC) ranks it as a promising substitute for existing dye removal techniques. The scalable production of AC can be ensured by using locally available waste biomass as raw precursor material, however the duration of treatment time, high temperature conditions and need for the continuous provision of a gaseous atmosphere could be possible reasons for the increased of these materials, compared to commercial AC. An efficient activated carbon that can be valorized at comparatively low cost, readily available locally and that can be regenerated easily are the most challenging characteristics demanded required by researchers.

In the current study, sawdust of widely available *Melia azedarach* was used as precursor raw material to produce AC on a laboratory scale using orthophosphoric acid as an activating agent. *Melia azedarach* is a locally grown woody plant mainly employed for obtaining hard lignocellulosic wood for making furniture and other construction industry-related items. The manufactured novel activated carbon was employed to treat an aqueous solution of reactive orange 16 (RO16) dye in batch reactors. The adsorbent material was characterized, and its surface charge and surface functional groups were determined. Surface area, pore volume and pore width diameter were also analyzed. Desorption and cost analyses of the studied treatment system were conducted to determine its suitability for field application.

## 2. Results and Discussion

### 2.1. Characterization of the MA-AC400 Adsorbent

#### 2.1.1. Scanning Electron Microscopy (SEM) and Energy Dispersive X-ray Spectroscopy (EDS)

**Figure 1:**
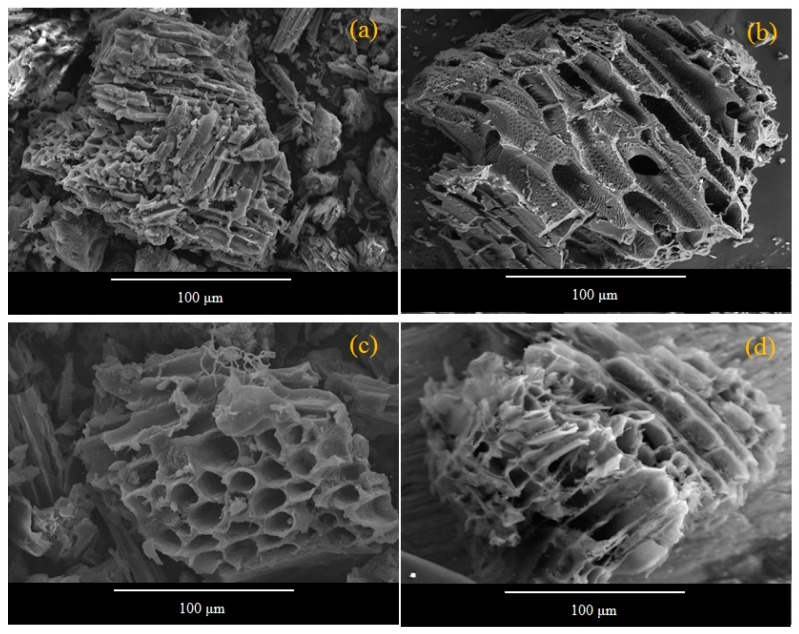
(**a**,**b**) SEM micro images of MA-AC400 adsorbent before RO16 adsorption; (**c**,**d**) after RO16 dye adsorption.

**Figure 2:**
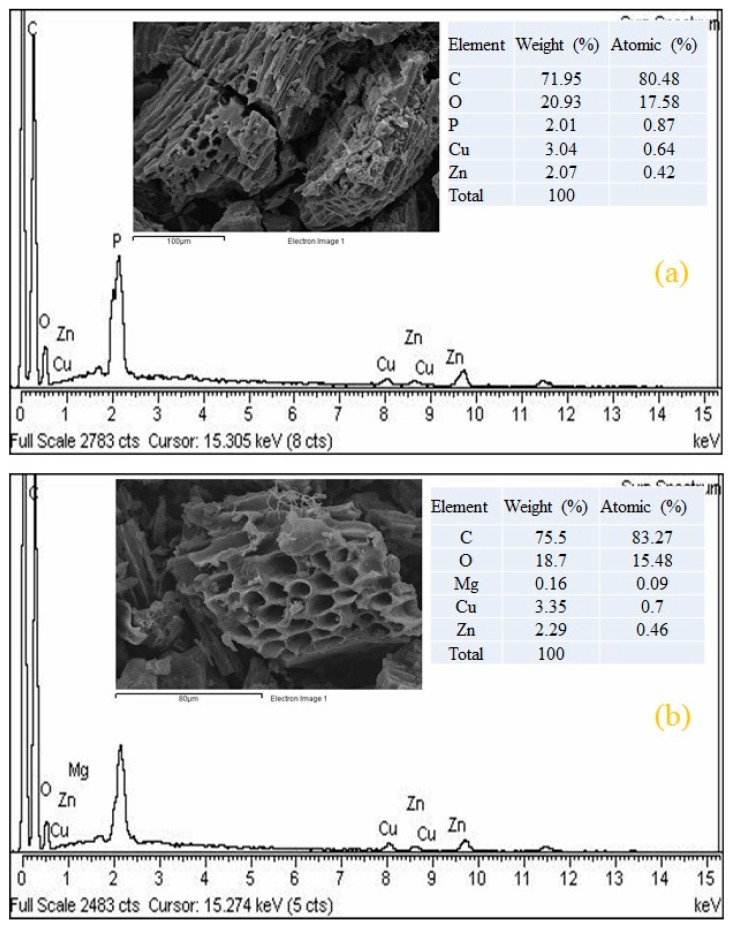
(**a**) Energy dispersive spectrum (EDS) of MA-AC400 before RO16 adsorption; (**b**) EDS of MA-AC400 after adsorption of RO16 dye.

The surface morphology of MA-AC400 has been explored by SEM before (Figure 1a,b) and after (Figure 1c,d) RO16 dye adsorption. It is obvious from the figures that MA-AC400 presents a well-developed porous structure and can be useful for the uptake of dye molecules either through a physical mechanism via diffusion, and/or a chemical mechanism via bonding at the active sites located within the pores of MA-AC40. Changes in the compositional makeup of MA-AC400 after adsorbing RO16 dye were detected through EDS analysis and can be witnessed in Figure 2b when compared with Figure 2a which shows the energy spectrum of MA-AC400 before dye loading. For instance a percent increase was observed in the weight and number of C atoms once the MA-AC400 interacted with the RO16 dye in the batch reactor. Besides this a percent decrease was observed in the weight and number of O atoms after loading the MA-AC400 with RO16. This affirms the interaction of chemical species of RO16 present in the aqueous phase and the adsorbent surface. MA-AC400 is an extra dense solid material offering plentiful craters (macro-pores) produced by the action of the orthophosphoric acid after it is removed from the AC during neutralization with distilled water, creating accessible pore structures [28]. Furthermore, MA-AC400 is a rich carbonaceous solid with rich C contents that appears to be a good candidate for the maximum adsorption of RO16 dye. This C-enrichment of MA-AC400 is probably due to the liberation of oxygen during the activation process [29].

#### 2.1.2. Fourier Transform Infrared Spectroscopy (FTIR) Analysis

**Figure 3:**
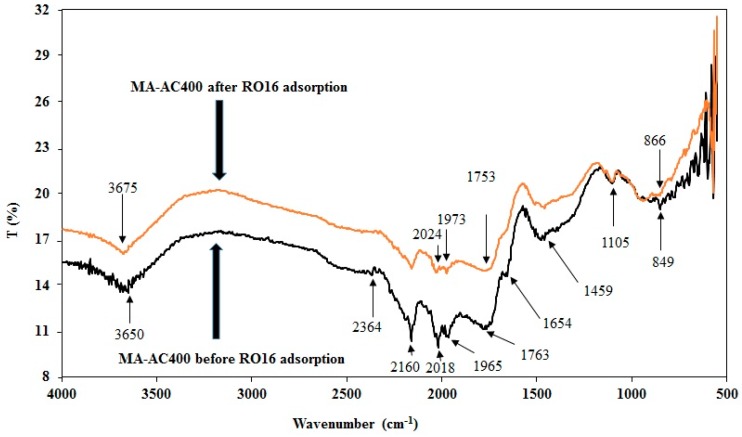
FTIR transmittance spectral analyses for MA-AC400 before and after RO16 loading.

The FTIR spectrum of MA-AC400 presented in Figure 3 reveals the presence of strong interactions between the RO16 dye constituents and the MA-AC400 adsorbent. All possible functional groups were identified with the help of the literature on AC production by H_3_PO_4_ activation of carbon. After carbonization of *Melia azedarach* waste sawdust at 400 °C, the FTIR peak at 849 cm^−1^ demonstrated the presence of C-H deformation for different aromatic ring substituents [30]. 

Importantly the band appearing at 1105 cm^−1^ can be ascribed to the incorporation of phosphorous groups within the carbon structure and the development of aromatic phosphates [31] and an increase of the phosphorous content in the AC [30]. MA-AC400 has transmission spectra bands which appear at 1459 cm^−1^, 1654 cm^−1^, 1763 cm^−1^, and 2160 cm^−1^ indicating the presence of medium C-H bending, weak C-H bending (aromatic compounds), strong C=O stretching (anhydride), and C≡C (alkyne group) stretching, respectively [28]. The peak around 2018 cm^−1^ appeared during H_3_PO_4_ activated thermal manufacturing of AC from wood biomass [32]. The medium peak found at 2364 cm^−1^ is associated to the C=C group. The spectral peak at 3650 cm^−1^ can be attributed to H_3_PO_4_ treatment, stretching O–H bonds in carboxylic acid functional groups, and adsorbed water [29,33]. An increase in the peak transmittance intensities in MA-AC400 samples loaded with RO16 dye was typically observed and the peak positions shifted from 849 cm^−1^, 1965 cm^−1^, 2018 cm^−1^, 2160 cm^−1^, 3650 cm^−1^ to 866 cm^−1^, 1973 cm^−1^, 2024 cm^−1^, 3675 cm^−1^, respectively, which revealed the interaction of the RO16 dye molecules and the MA-AC400 adsorbent. Moreover spectral peaks at 1654 cm^−1^ and 2364 cm^−1^ also confirm the participation of C-H and C=C functional groups in molecular bonding after RO16 dye adsorption. The above observations manifest the obvious binding interactions of RO16 dye molecules with the MA-AC400 adsorbent.

#### 2.1.3. Dynamic Light Scattering (DLS) Analysis

**Figure 4:**
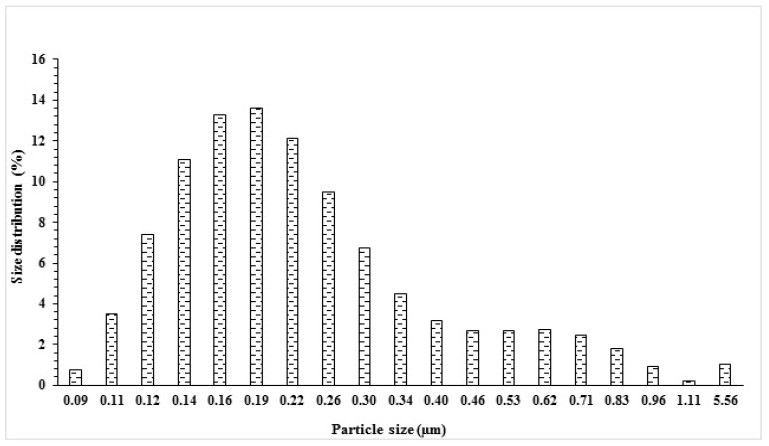
The size distribution of MA-AC400 particles in aqueous solution using dynamic light scattering.

In the dynamic light scattering analysis, MA-AC400 adsorbent was seemingly composed of large grain sizes. Sonication (6 h, 100 W power, FSF-020S, Ultrasonic, China) was applied to 20 mg of the adsorbent and the adsorbent was dispersed in water. The latter converts the large aggregates of adsorbent once in the aqueous phase into finer particle sizes. Figure 4 demonstrates the DLS results of MA-AC400 adsorbent in an aqueous solution comprising 200 mL of distilled water and 20 mg of the MA-AC400. The DLS analyses was intended to determine the particle size distribution in the solid-liquid mixture. The results displayed the particle size distribution was in the 0.09–5.56 µm range with an average size of 0.69 µm, however the majority of the particle sizes were observed in a narrow range of 0.12 to 0.30 µm, as can be seen from Figure 4. A similar analysis has been reported elsewhere for H_3_PO_4_-activated carbon produced from rice husk [34].

#### 2.1.4. Fluorescence Analysis of MA-AC400

**Figure 5:**
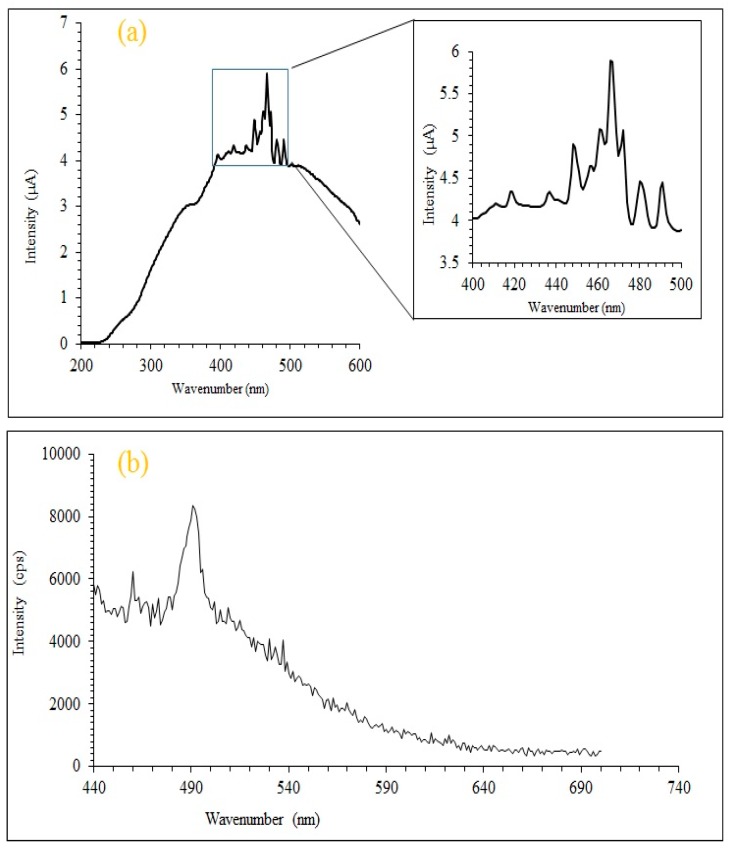
(**a**) Full range excitation spectrum of MA-AC400 (400–500 nm); (**b**) emission spectrum of MA-AC400 excited at 420 nm.

A full range wavelength excitation spectrum was recorded in the UV-Visible range from 200 nm until 600 nm for an aqueous mixture containing MA-AC400 adsorbent (Figure 5a). It can be seen from the inset in Figure 5a that the spectrum exhibits multiple excitation wavelength peaks, for instance at 420, 437, 448, 458, 462, 472, 481 and 491 nm. These multiple lower energy excitation peaks reflect the presence of different sizes of MA-AC400 particles in the aqueous phase. This polydispersity was also observed in the DLS results (Figure 4). Afterwards the emission spectrum was recorded at 420 nm of excitation wavelength and the maximum emission intensity is observed at a relatively shorter emission wavelength, i.e., 497 nm in the visible region (Figure 5b).

#### 2.1.5. Textural Characterization of MA-AC400

The textural characteristics of MA-AC400 adsorbent are summarized in Table 1. The significance of laboratory scale manufactured MA-AC400 is evident with high BET (S_BET_ = 293.12 m^2^ g^−1^) and Langmuir surface area (440.83 m^2^ g^−1^). This enormous surface area can accommodate a hefty amount of dye molecules to be adsorbed expediently. Pore size distribution is a very important trait of any porous adsorbent since differences in the pore size significantly affect the adsorption capacity of contaminant molecules of dissimilar sizes and geometrical forms [35]. The abundant availability and diverse pore size distribution on the surface of MA-AC400 depicted in Table 1 ensures the availability of possible binding sites for RO16 dye molecules present in the aqueous medium [36]. The BJH average pore diameter calculated was found to be 2.136 nm, which categorized MA-AC400 as a mesoporous material [37]. This advantageous mesoporous structure of MA-AC400 augmented its effectiveness for attracting the large sized RO16 dye molecules. This dependence on meso-porosity of adsorbents’ capabilities has been demonstrated for the adsorptive removal of analogous dye molecules such as methylene blue, rhodamine B on activated carbons [38].

### 2.2. Batch Reactor Adsorption Studies

#### 2.2.1. Effect of Contact Time

**Figure 6:**
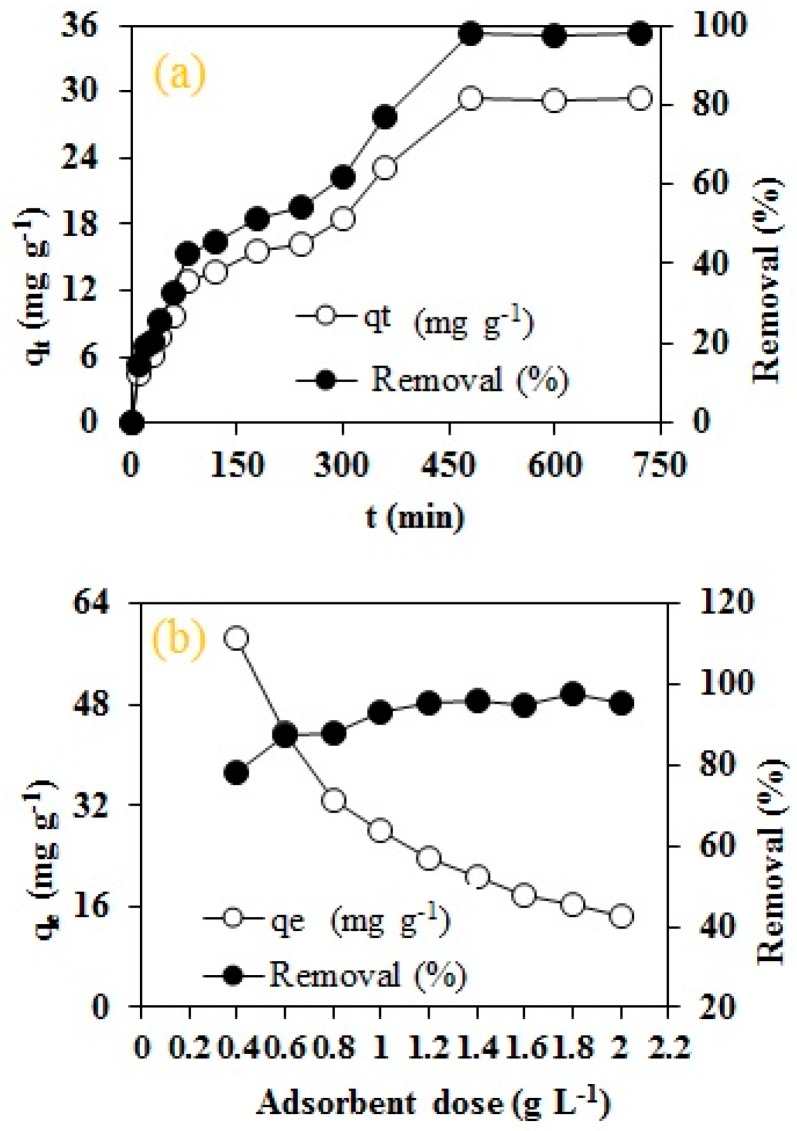

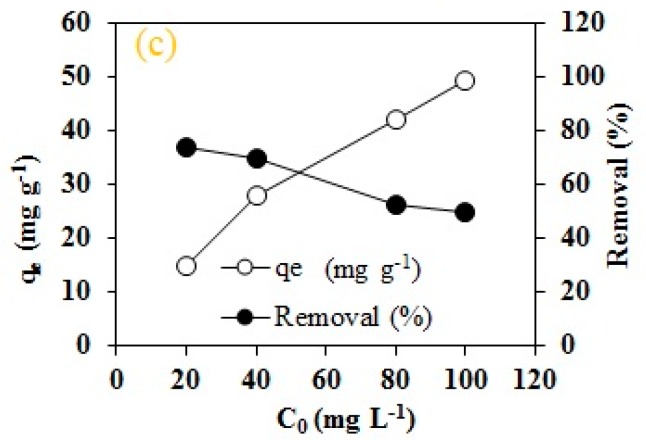
Effect of batch process variables on adsorption of RO16 by MA-AC400: (**a**) contact time; (**b**) adsorbent dose; (**c**) initial concentration of dye.

The establishment of an adsorption equilibrium is one of the key factor while designing an adsorption technique for a full scale treatment facility. The effect of contact time on RO16 dye adsorption by the novel MA-AC400 was investigated at time durations ranging from 10 min to 720 min, as shown in Figure 6a. It is quite evident from the figure that initially the RO16 uptake by MA-AC400 was quite instantaneous. The overall RO16 dye uptake mechanism can broadly be classified into three distinct phases: (i) rapid uptake of 42% of the dye during the first 80 min; (ii) a sluggish progression of RO16 uptake from 80 to 480 min reaching 97.8% removal and (iii) the adsorption progress becomes negligible in the last stage from 480 to 720 min. During first phase, the adsorbent possess a huge number of free available sites for the binding of dye molecules however with the passage of time, the availability of these sites becomes sporadic thus explaining the declining efficiency. 

The decrease in dye uptake can be attributed to the fact that the competition of dye molecules present in the aqueous phase increases due to the fewer available binding sites. Furthermore, the sluggish progression of RO16 adsorption during the second stage might be due to a prevalent intraparticle diffusion adoption mechanism. The same kind of trend have been observed by many other researchers when investigating adsorption processes using walnut hulls [39], pistachio hulls [40], orange peel [41], banana peel [42] and pineapple waste biomass [42].

#### 2.2.2. Effect of Adsorbent Dose

The quantity of the adsorbent plays a pivotal role in governing the overall performance of any adsorption process. The RO16 dye uptake from aqueous solution depends on the dosage of MA-AC400 as shown in Figure 6b. It is apparent that by increasing the amount of adsorbent (0.4 to 2 g L^−1^), the RO16 removal jumped from 78 to 97.59%, respectively. This trend can be attributed to the availability of numerous binding sites on the surface of MA-AC400. Thus, a higher quantity of dye molecules find their way to be adsorbed by the adsorbent being exposed during the contact phase. Similar kinds of trends have been reported for RO16 dye using TiO_2_ nanocomposites [43], chitosan/sepiolite composites [44], and fish scales [45]. However, the adsorption capacity (mg g^−1^) declined significantly from 58.54 to 14.32 mg g^−1^ with the increase in MA-AC400 from 0.4 to 2 g L^−1^ respectively. The fall in adsorption capacity can be accredited to accumulated dye molecules on the surface of MA-AC400 coupled with an increase in the path distance for effective diffusion [46].

#### 2.2.3. Effect of Initial Dye Concentration

The aptitude of MA-AC400 to eliminate RO16 at different initial concentration levels (20 to 100 mg L^−1^) was investigated and is presented in Figure 6c. The percentage removal of RO16 by MA-AC400 was found to be decreased from 73.56 to 49.23% by increasing the dye concentration from 20 to 100 mg L^−1^. On the other hand, the adsorption capacity enhanced from 49.23 mg g^−1^ as the initial dye concentration was changed five times. The dye uptake per unit mass of adsorbent increased due to the available vacant binding sites on the surface of MA-AC400. The decreasing dye percentage can be ascribed to the fact that a higher number of dye molecules in aqueous phase leads to competition and conglomeration at the adsorbent surface offering resistance the mass transfer.

#### 2.2.4. pH, pH_pzc_ and Boehm Titration Analyses

**Figure 7:**
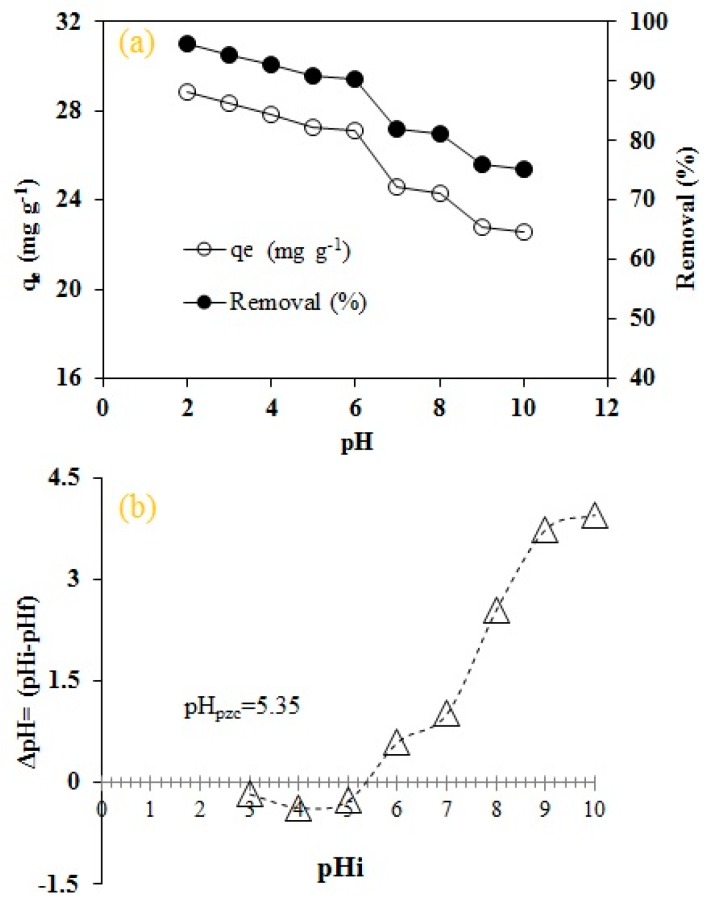
Effect of batch process variables on adsorption of RO16 by MA-AC400: (**a**) pH; (**b**) pH_pzc_.

RO16, being an anionic dye, has strong affinity towards positively charged surfaces for effective adsorption. It can be seen from Figure 7a that the maximum dye uptake (96.27%) was observed at pH 2 which declined to 75.20% under highly alkaline conditions (pH 10). This decrease can be attributed to the repulsive electrostatic interaction between anionic dye groups and hydroxyl ions present in liquid phase. The protonated functional groups on the surface of MA-AC400, including carboxylic acid (-COOH_2_^+^), and phenolic groups (-OH_2_^+^) and the deprotonated sulphonate groups (-SO_3_^−^) interacted with each other resulting in higher dye uptake [47,48,49,50]. The dye removal efficiency remained stable in the 4–6 pH range due to protonated sulphonic acid groups which act as neutral molecules [51]. Similarly, the dye removal declined with increasing alkalinity supplementing the deprotonated dye molecules coupled by hydroxyl ions in aqueous medium. An identical drift of increasing reactive dye uptake by banana and orange peels [41], AC from Brazilian pine fruit shells [52], AC from *Enteromorpha prolifera* [47], AC loaded with cationic surfactant [53] and orange waste [54] has been confirmed by other researchers thus affirming our current findings.

The adsorbent surface charge plays a pivotal role in affecting the adsorption mechanism [55]. Figure 7b shows the adsorbent surface charge neutrality at pH 5.35 which indicates the prevalence of positive surface charges when the solution pH < pHpzc and negatively charged pH > pHpzc, respectively [56]. RO16 dye being an anionic dye is overwhelmingly attracted by the surface of MA-AC400 under acidic conditions as described above, followed by a decrease under alkaline conditions [57]. The isoelectric nature of adsorbent surface and dye molecules under alkaline conditions results in repulsive interactions hindering the maximum dye uptake from aqueous solution [58].

Oxygen-containing functional groups present on MA-AC400 surface were analyzed by “Boehm” titration. The results demonstrated more acidic than basic groups, confirming the acidic character of MA-AC400. This finding reinforces the RO16 removal under acidic pH, and pH_pzc_ value of the adsorbent (Figure 7a,b). The total oxygenated acidic functional groups in MA-AC400 were 3.5 mmol g^−1^ while the total basic groups were 0.45 mmol g^−1^. The predominant oxygenated group distribution was phenolic (1.7 mmol g^−1^), carboxylic acid (0.30 mmol g^−1^) and lactonic (1.5 mmol g^−1^). The dominance of phenolic groups was also reported by previous studies in which activation was achieved by H_3_PO_4_ at 450 °C [59,60].

#### 2.2.5. Effect of Temperature

**Figure 8:**
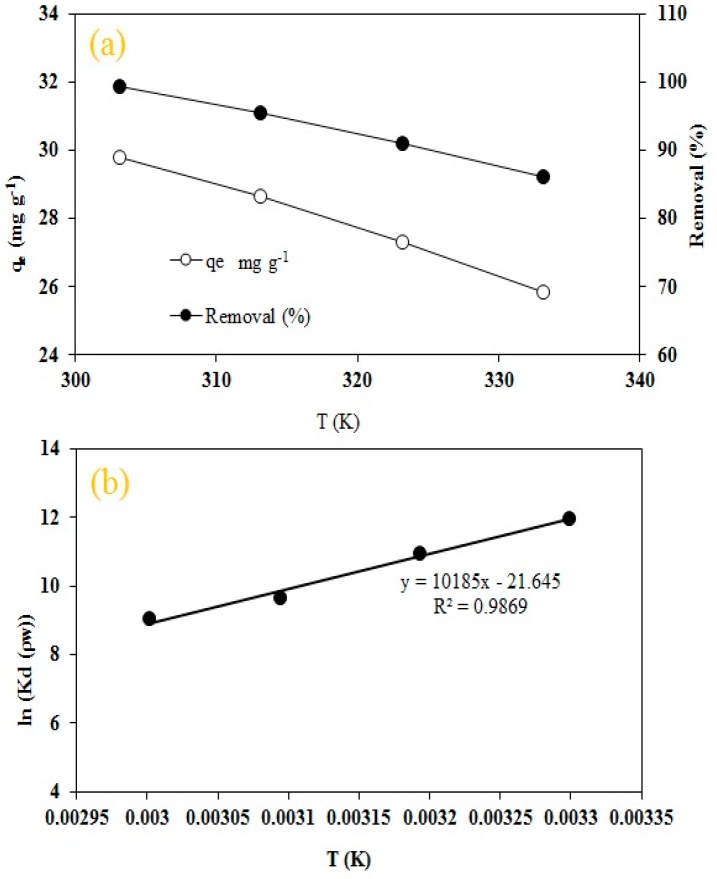
(**a**) Effect of temperature on MA-AC400 forRO16 adsorption; (**b**) Van’t Hof plot.

The impact of temperature on RO16 dye adsorption by MA-AC400 was examined at four different temperatures (303.15, 313.15, 323.15 and 333.15 °C) at a solution pH of 6.2, 150 rpm agitation speed, 30 mg L^−1^ initial dye concentration and an adsorbent dosage of 1 g L^−1^. The results are portrayed in Figure 8a which is displays an inverse relationship between RO16 dye removal and temperature, whereby the dye uptake declined from 99.37 to 86.17%. This phenomenon affirms the endothermic nature of the adsorption process during dye uptake by MA-AC400.

The parameters calculated for RO16 adsorption by MA-AC400 under variable temperatures are summarized in Table 2 (Figure 8b). The negative value of ΔH characterizes an exothermic adsorption process. The positive ΔS indicates the favorable, irreversible stability and some structurally-based dye-activated carbon complexes during the adsorption process coupled by an increased state of randomness at the solid/liquid interface [61]. The cumulative ΔG with rising temperature demonstrates its favorability. The negative ΔG results suggested a spontaneous adsorption process of RO16 by MA-AC400. Additionally, ΔG values were within the range of −139.23 to −144.63 kJ mol^−1^, indicative of a chemisorptive adsorption mechanism which is consistent with findings of a pseudo-second order kinetics supported by high coefficient of determination for Langmuir isotherm in the current adsorption system [62].

### 2.3. Mechanism of RO16 Adsorption onto MA-AC400

#### 2.3.1. Equilibrium Isotherm Analysis

**Figure 9:**
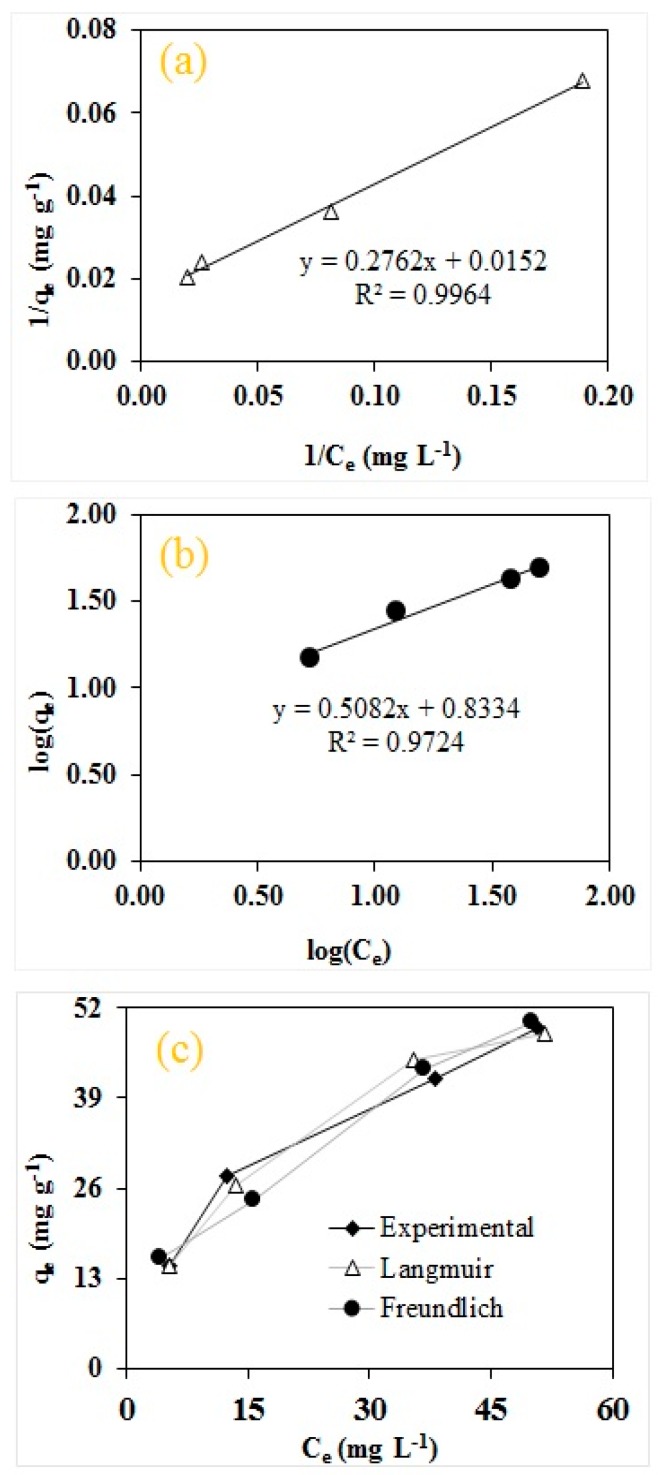
Isotherm plots for RO16 adsorption onto MA-AC400: (**a**) Langmuir; (**b**) Freundlich; (**c**) deviation of predicted adsorption capacities calculated by Langmuir and Freundlich isotherms with the experimental value.

The adsorption process for RO16 by MA-AC400 was better explained in order of Langmuir < Freundlich in view of the coefficient of determination (R^2^) as shown in Figure 9. The Langmuir adsorption capacity was found to be 65.79 mg g^−1^ with R^2^ of 0.9964. Moreover the value of K_L_ was found to be 0.06, which confirms the chemical interaction between RO16 and MA-AC400 adsorbent. Although R^2^ obtained for the Freundlich isotherm fit was found low, i.e., 0.9724 compared to Langmuir yet the value Freundlich constant (1/n) was observed to be less (0.51 in this study) than 1, implying the existence of physisorption of RO16 onto the MA-AC400 adsorbent which reveals the co-existence of some heterogeneous surfaces on MA-AC400. Moreover KF and n were found to be 6.81 mg g^−1^ and 1.97, which showcase the positive cooperation and favorability of MA-AC400 for RO16 adsorption.

Error analysis is carried out to validate and reinforce the correctness of adsorption data fitness to applied isotherms and the respective predictions of models. The smaller the error values for each isotherm, the larger the goodness of fit for the adsorption data to a particular isotherm except for R^2^ which indicates high goodness of fit if its value is closer to unity and vice-versa. In this study, five types of error functions, including the R^2^, χ^2^, SAE, MPSD and HYBRID, were calculated taking into consideration the experimental adsorption capacities (q_e exp_) for RO16 dye by MA-AC400 and the values predicted/calculated (q_e cal_) from the linear isotherm equations. It can be seen from Table 3 that values determined for all error functions were observed to be smaller for Langmuir than for the Freundlich isotherm while the R^2^ value was found closer to unity for the Langmuir isotherm compared to the Freundlich one. It can be concluded from the above discussion that RO16 dye adsorption on MA-AC400 is better described by a Langmuir isotherm and the adsorbent possesses a more homogenous surface with equally distributed adsorption sites. Likewise to the error function analysis, Langmuir prediction reveals less deviation of the adsorption capacities from the experimental adsorption of MA-AC400 in comparison to the Freundlich isotherm (Figure 9c). This also indicates that adsorption dataset is better described by a Langmuir isotherm compared to the Freundlich one.

#### 2.3.2. Adsorption Kinetics

**Figure 10:**
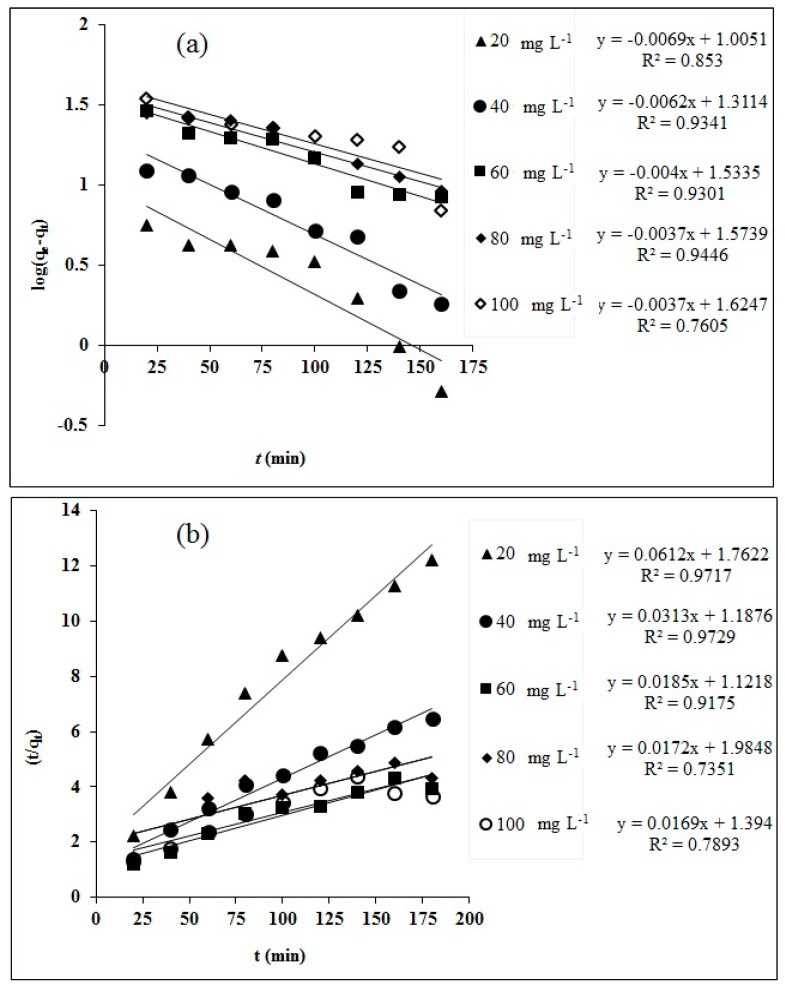
Linear kinetic fitness for RO16 adsorption onto MA-AC400: (**a**) PFO kinetic plot; (**b**) PSO kinetic plot.

The plots of PFO and PSO order kinetic models are presented in Figure 10a,b and the respective parameters and constant are listed in Table 4. Higher deviation of q_e cal_ from q_e exp_ and lower R^2^ values were observed for the PFO model at lower initial concentration (C_0_, mg L^−1^) of RO16 compared to PSO and the opposite was found true at higher C_0_ for PFO than that of PSO. This indicates the PSO kinetics best explain the adsorption process of RO16 dye by MA-AC400 at lower C_0_ and the reverse occurs for PFO. These findings are reinforced in view of the R_2_ values as well. Moreover it also reveals that the chemical interaction is strong at lower C_0_ and some physical forces, e.g., concentration gradient and diffusion phenomenon at higher concentration of RO 16 dye in solution phase. The parametric constants, i.e., k_1_ and k_2_ for the PFO and PSO kinetic models, respectively, were plotted against the initial RO16 dye concentration and a declining trend was observed. This tendency suggests that the adsorption system with low contaminant concentration reaches its definite sorption capacity rapidly [63,64].

**Figure 11:**
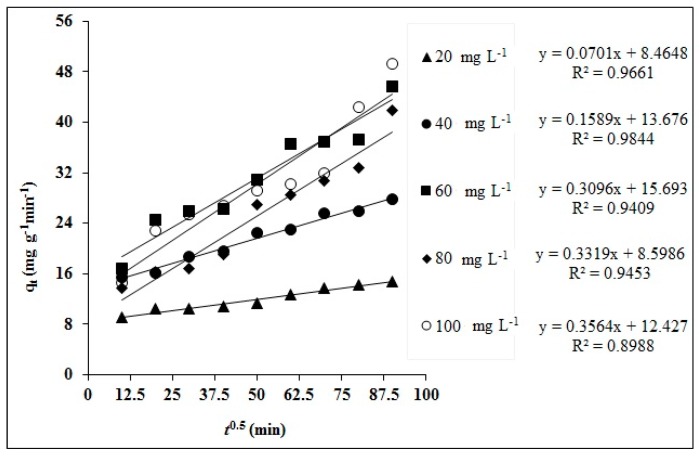
Intraparticle diffusion linear kinetic fitness plot for RO16 adsorption onto MA-AC400.

Intraparticle diffusion model (IPD) was chosen to uncover the diffusion mechanism of dye uptake by MA-AC400 (Figure 11). 

A linear plot of q versus t^1/2^ yields C (mg g^−1^) and K_pi_ (mg g^−1^ min^−1/2^) from the intercept and slope, respectively. The larger C indicates a higher contribution of the surface sorption as the rate limiting step. The good coefficient of determination (> 0.94) of the IPD model at both dilute and concentrated initial dye aqueous solutions confirms that his model explains well the adsorption phenomenon. The increasing Kpi values show the involvement of diffusion processes in RO16 dye uptake by MA-AC400 with rising dye concentration. The non-passage of the curve through the origin confirms that a diffusion mechanism is obviously involved but it is not the sole rate-limiting step.

### 2.4. Desorption Study of MA-AC400

**Figure 12:**
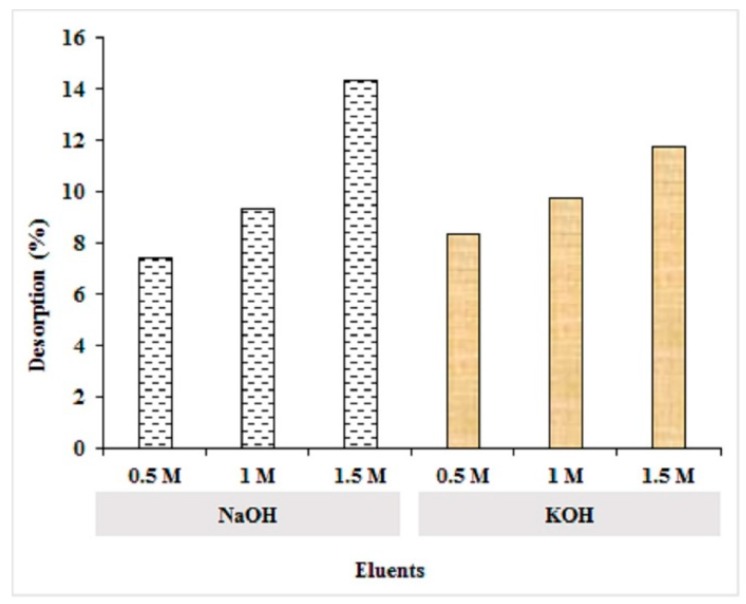
Desorption of MA-AC400 adsorbent.

The reutilization of adsorbent is an imperative consideration for practical application of adsorption at a large scale and alkaline solutions were chosen for desorption purposes because a lower adsorption of RO16 was observed at alkaline pH values (pH = 10, Figure 7a). The recycling of MA-AC400 was assessed and the results are depicted in Figure 12. It can be observed that increasing strength of alkaline solutions of KOH and NaOH resulted in increasing dye % desorption and regeneration of the adsorbent surface. The highest dye desorption i.e., 14.36% was observed for 1.5 M NaOH followed by 11.74% using 1.5 M potassium hydroxide as eluent. This phenomenon can be attributed to the fact that, under alkaline conditions, the deprotonation of amine groups at the surface of the adsorbent results in production of a negatively charged interface [44]. These identical anionic charges at the adsorbent surface and the dye constituents yields a repulsive interaction enhancing the desorption phenomenon [65].

### 2.5. Cost per kg of MA-AC400

In present study, removal of RO16 from aqueous solution has been explored by employing *Melia azedarach*-derived, orthophosphoric acid-activated carbon under optimized conditions. Table 5 summarizes the overall costs involved during the production process of laboratory scale MA-AC400. It is noteworthy that the total cost involved during preparation of MA-AC400 is 1388.33 PKR or $8.34 US/kg. The overall production cost/kg of MA-AC400 can be reduced if the manufacturing is based on a large facility and reclaiming H_3_PO_4_ during washing step. Moreover, any manufacturer’s profits, and marketing charges were not considered in the current cost analysis.

## 3. Materials and Methods

### 3.1. Chemicals and Reagents

Orthophosphoric acid (H_3_PO_4_), nitric acid (HNO_3_), sodium hydroxide (NaOH) and reactive orange 16 (RO16) dye were purchased from Sigma Aldrich (Darmstadt, Germany) and used without further purification. RO16 dye selected as a model pollutant has the key properties listed in Table 6. A stock solution of RO16 dye was prepared by dissolving an appropriate amount of dye in 1000 mL of deionized water. Working as well as standard solutions for calibration curve generation were prepared by subsequent dilutions.

### 3.2. Preparation of Activated Carbon (AC)

Sawdust of *Melia azedarach* was acquired from a local market situated in Hazara (Abbottabad, Pakistan). The biomass was ground in a lab scale crusher to obtain uniform particle size (40–60 mesh size). Powdered material was washed multiple times with deionized water to remove any adhered impurities. The powdered biomass was then oven dried at 105 °C until no further weight loss was observed followed by packing in airtight containers for further use. The activated carbon preparation process was accomplished in two phases: (i) the first phase involved the mixing of H_3_PO_4_ (conc.) with raw powdered material having impregnation ratio of 2 in an orbital shaker at 30 °C while an agitation speed of 150 rpm was maintained for 2 h. The homogenized mixture was then exposed to modest heat stress (100 °C) inside ac muffle furnace under inert atmosphere of N_2_ for 12 h; (ii) in the second phase, the material was exposed to a high temperature (400 °C) for 30 min under a CO_2_ atmosphere. The obtained activated carbon was thoroughly washed with deionized water until neutralization of residual acid was achieved (pH > 6.5). Thereafter the produced carbonaceous material was oven dried at 105 °C until a constant weight was achieved and it was labelled as MA-AC400.

### 3.3. Characterization of MA-AC400

Infrared spectra of the MA-AC400 were obtained using a Fourier transform infrared spectrometer (Alpha Bruker, Karlsruhe, Germany). For FT-IR study, ground MA-AC400 was gently mixed with potassium bromide (KBr) to get a translucent pelletized sample and FTIR spectra runs were carried out in the 500 to 4000 cm^−1^ wavenumber range. The surface morphology and elemental composition of the MA-AC400 was examined using a scanning electron microscope (SEM) equipped with an energy dispersive X-ray spectroscopy (EDS) system using a JEOL JSM 5910 instrument (INCA 200, Oxford Instruments, High Wycombe, UK). Dynamic light scattering (DLS) measurements were performed at 25 °C in water on a Zetasizer Nano ZSP from Malvern Instruments Limited (Malvern, UK) using the 173° angle non-invasive backscatter mode and the M3-phase analysis light scattering mode, respectively. The instrument has a red 10.0 mW 633 nm He−Ne laser. The accuracy was better than ±2% on NIST-traceable latex standards. The multiple peak high-resolution fitting procedure was used to obtain the particle size distribution from the autocorrelation function. Intensity distribution curves for AC were considered for particle size distribution. Measurements were performed with AC at 0.1 mg mL^−1^ concentration. Fluorescence spectroscopy was performed at room temperature on a Fluoromax-4 spectrophotometer (Horiba, NY, USA). Measurements were taken by using the quartz cuvette with dimensions 10 × 10 mm, transparent from all sides. The slit width was fixed at 3 mm with a band pass of 12.75 nm. 0.1 mg mL^−1^ of as synthesized AC carbon was dispersed in distilled water to measure the fluorescence using a 50 Hz ultrasonicator (FSF-020S, Huanghua Faithful Instrument Co., LTD, Huanghua, Hebei Province, China) for 6 h.

#### 3.3.1. pH_PZC_ Determination and Boehm Titration

For pH at point of zero charge (pH_pzc_) determination [22], the surface charge distribution was analyzed by addition of 25 mg MA-AC400 adsorbent to 50 mL of 0.1 M NaCl solution and the pH was adjusted between 3–10 by addition of 0.1 M HCl and NaOH solutions. These mixtures were sealed and agitated at 150 rpm for 24 h under 30 °C. After separation, the final pH of each solution was measured quickly. pH_PZC_ corresponds to the net electrical neutrality of the adsorbent obtained from the intersection point by plotting ${pH}_{i}\;vs.\;\Delta pH\;or\;({pH}_{f}-{pH}_{i})$ [66].

#### 3.3.2. Textural Properties of MA-AC400

The Boehm titration method [67] was employed for the determination of various acidic/basic functional groups distributed onto the surface of MA-AC400 adsorbent. Briefly 1.0 g of MA-AC400 and 15 mL solution of NaHCO_3_ (0.1 M), Na_2_CO_3_ (0.05 M) and NaOH (0.1 M for acidic groups) and 0.1 HCl for basic group/sites, respectively, were kept at room temperature for 2 days. Later, the aqueous solutions were back titrated with NaOH (0.1 M) and HCl (0.1 M) for basic and acid groups, respectively. The type and number of acidic groups were calculated by bearing in mind that NaOH neutralizes carboxylic acid, lactonic and phenolic groups while Na_2_CO_3_ neutralizes carboxylic acid and lactonic groups and that NaHCO_3_ neutralizes only carboxylic acid groups. The quantity of functional groups containing oxygen, F_x_, is calculated using Equations (1) and (2):

(1) $$F_{x}=\frac{\left(V_{\mathrm{bx}}-V_{\mathrm{ex}}\right)}{m_{x}}\times M_{t}\times \mathrm{DF}$$

(2) $$\mathrm{DF}=\frac{\mathrm{initial}\;\mathrm{volume}}{\mathrm{Selected}\;\mathrm{volume}\;\mathrm{for}\;\mathrm{titration}}$$

where F_x_ (mmol g^−1^) V_bx_, V_ex_, M_t_ and DF are the total oxygen-containing functional groups, volume of titrant used to titrate the blank, volume of the titrant being used to titrate the extract, molarity of the titrant used and dilution factor, respectively.

The textural properties of MA-AC400 were evaluated through frequently adopted nitrogen adsorption-desorption isotherm analysis at 77.30 K using a GEMINI VII 2390 surface area analyzer (Micromeritics Instrument Corp, Norcross, GA, USA). The Brunauer–Emmett–Teller (BET) method was adopted to calculate the specific surface areas (S_BET_). Additionally, the micropore surface area (S_micro_) and micropore volume (V_micro_) were calculated by De Boer’s t-plot method. The external surface area (S_external_) was calculated as the difference between SBET and S_micro_. The total pore volume (V_total_) was estimated in terms of the amount of nitrogen gas adsorption at a relative pressure (P/P_0_) of 0.98 by using the Horvath–Kawazoe method [68]. The micropore area and volume were calculated using the t-plot method [69].

### 3.4. Batch Reactor Studies

Batch adsorption investigations were conducted in aqueous phase mixtures of MA-AC400 and RO16 dye and all adsorption studies were performed at 30 ± 2 °C. A series of 50 mL Erlenmeyer flasks containing 20 mL of RO16 solution having variable initial concentrations of 20 to 100 mg L^−1^ and MA-AC400 dosage of 0.40 to 2 g L^−1^ were employed at a chosen pH of 2 to 10 and pH were adjusted by adding 0.1 M HCl or 0.1 M NaOH solutions. These flasks were shaken using shaker equipped with digital fuzzy control system (Wise Cube, Seoul, Korea) at 150 rpm for a specific period of contact time (10–600 min) in order to achieve equilibration time. The filtrates were then analyzed using UV-Visible double beam spectrophotometer (T80^+^ PG instruments, Leicestershire, UK) at λ_max_ = 492 nm under calibrated conditions. The adsorption capacities of MA-AC400 at time t (q_t_, mg g^−1^) and at equilibrium time (q_e_, mg g^−1^) and percentage RO16 dye removal of MA-AC400 were calculated by employing the following Equations (3)–(5):

(3) $$q_{t}=\left(\frac{C_{0}-C_{t}}{m}\right)\;V$$

(4) $$q_{e}=\left(\frac{C_{0}-C_{f}}{m}\right)\;V$$

(5) $$\mathrm{Removal}\;\left(\%\right)=\left(\frac{C_{0}-C_{f}}{C_{0}}\right)\;100$$

where V (L), m (g), C_0_, C_t_, and C_f_ are the volume of solution, mass of adsorbent, initial, at any time “t” and final RO16 dye concentration in aqueous solution (mg L^−1^), respectively.

### 3.5. Thermodynamic Studies

Thermodynamic parameters were calculated to evaluate thermodynamically the nature of adsorption in the RO16 and MA-AC400 system. The thermodynamic equilibrium constant was calculated using Equation (6). To make it dimensionless, this equilibrium constant was multiplied by solution (water) density (ρw ≈ 1000 g L^−1^) [22,70,71]. Changes in enthalpy ΔH (kJ mol^−1^) and entropy ΔS (J mol^−1^ K^−1^) were obtained via intercept and slope of Van’t Hoff plot lnKd(ρw) against 1/T (Equation (7)) and finally the Gibb’s free energy ΔG (kJ mol^−1^) was determined using Equation (8):

(6) $$l_{n\;}K_{d}\left(\rho_{w}\right)=\frac{q_{e}}{C_{e}}$$

(7) $$\ln K_{d}\left(\rho_{w}\right)=\frac{\Delta S}{R}-\frac{\Delta H}{\mathrm{RT}}$$

(8) ΔG=ΔH−TΔS

### 3.6. Isotherm Studies

Adsorption isotherm models are mathematical tools extensively employed to designate the type of molecule coverage, adsorbent surface homogeneity/heterogeneity, binding energies, physical/chemical nature of interaction and adsorption heat of molecules. The equilibrium data of RO16 dye on MA-AC400 was evaluated using linearized forms of the widely applied Langmuir and Freundlich models and the results of parameters and constants were calculated. The Langmuir isotherm [72] assumes a monolayer coverage of adsorbate molecules onto the surface of an adsorbent predominantly via chemical interaction. The Freundlich isotherm [73] is generally applied to heterogeneous surfaces assuming a multilayer adsorption of adsorbate molecules onto the surface of adsorbent and binding sites are not equally distributed. The linearized forms of the Langmuir and Freundlich isotherm models are given by Equations (7) and (8), respectively:

(9) $$\frac{1}{q_{e}}=\frac{1}{q_{e\;cal}}+\left(\frac{1}{q_{e\;\mathrm{cal}}K_{L}}\right)\frac{1}{C_{e}}$$

The linear plot of 1/q_e_ vs. 1/C_e_ yields Langmuir parameters of calculated q_e cal_ (mg g^−1^) and KL (L mg^−1^). KL measures the adsorption affinity of adsorbate (RO16 dye molecules) to the adsorption sites of adsorbent, i.e., MA-AC400 (in current study). Freundlich isotherm can be described as follows:

(10) $$\log\;\left(q_{e}\right)=\log\;(k_{f})+\frac{1}{n}\log\;\left(c_{e}\right)$$

where KF is the Freundlich constant which is related to the affinity between interacting species and 1/n measures the nature of the adsorption for which the heterogeneity increases as the value gets nearer to zero [74]. KF and 1/n can be determined from intercept and slope of plot log (q_e_) versus log (C_e_) respectively.

Closeness between predicted adsorption by both linear models and experimental adsorption of MA-AC400 for RO16 was determined through normalized standard deviation, which is expressed as:

(11) $$\Delta q\;\left(\%\right)=100\;\sqrt{\frac{\left(q_{t\;\exp}-q_{t\;\mathrm{cal}}\right)}{n-1}}$$

#### Error Function Analysis for Linear Isotherms

Linear regression is one of the distinct and widely adopted statistical tools employed for connecting and validating experimental and modeled data in adsorption studies. These error functions are widely employed to cross-examine the findings of mathematical, empirical and semi-empirical models with those of experimental findings in order to maintain consistency and credibility of scientific data. Numerous research studies have revealed that the error range of experimental data is generally altered throughout the conversion of adsorption isotherms into forms after linearization [75]. In our current work, five different error functions including coefficient of determination (R^2^) [22], chi-square error (χ^2^) [76], sum of absolute error (SAE) [77], Marquardt’s percent standard deviation (MPSD) [78] and HYBRID functional error (HYBRID) [79] were calculated and the goodness of fit for Langmuir and Freundlich was suggested. All error functions are represented by Equations (12)–(16):

(12) $$R^{2}=\frac{\sum {\left({q_{t}}_{\mathrm{cal}}-{{\bar{q}}_{t}}_{\exp}\right)}^{2}}{\sum {\left({q_{t}}_{\mathrm{cal}}-{{\bar{q}}_{t}}_{\exp}\right)}^{2}+\sum {\left({q_{t}}_{\mathrm{cal}}-{q_{t}}_{\exp}\right)}^{2}}$$

(13) $$\chi^{2}=\sum_{i=1}^{n}\frac{{\left(q_{e\;\exp}-q_{e\;\mathrm{cal}}\right)}^{2}}{q_{e\;\mathrm{cal}}}$$

(14) $$\mathrm{SAE}=\sum_{i=1}^{n}\left|q_{e,exp}-q_{e,cal}\right|$$

(15) $$\mathrm{MPSD}=100\sqrt{\frac{1}{n-p}\sum_{i=1}^{n}{\left(\frac{q_{e\;\exp}-q_{e\;\mathrm{cal}}}{q_{e\;\exp}}\right)}^{2}\;}$$

(16) $$\mathrm{HYBRID}=\frac{100}{n-p}\sum_{i=1}^{n}{\left(\frac{q_{e\;\exp}-q_{e\;\mathrm{cal}}}{q_{e\;\exp}}\right)}^{2}\;$$

### 3.7. Kinetic Modelling

Adsorption kinetic studies are crucial in the optimization of adsorption process conditions for contaminant adsorption. The adsorption kinetics of RO16 dye were investigated using three widely employed models, namely the pseudo-first order (PFO), pseudo-second order (PSO) and intraparticle diffusion model (IPD), which can be represented in Equations (17)–(19). Adsorption kinetics were studied at 20, 40, 60, 80 and 100 mg L^−1^ of initial dye concentrations over a time duration of 180 min:

(17) $$\log(q_{e}-q_{t})=\log q_{e}-k_{1}t/2.303$$

(18) $$\frac{t}{q_{t}}=\frac{1}{K_{2}q_{e}^{2}}+\frac{1}{q_{e}}t$$

(19) $$q_{t}=K_{\mathrm{pi}}t^{\frac{1}{2}}+C_{i}$$

Here K_1_, K_2_ and K_pi_ represent PFO, PSO and IPD constants. C_i_ determines the boundary layer thickness.

### 3.8. Desorption Study

To investigate the reusability of spent MA-AC400, a desorption study was performed. In brief, RO16-loaded MA-AC400 adsorbent was oven dried and added into a 50 mL flask. Eluents, namely 0.5, 1.0 and 1.5 M strength solution of 30 mL of each sodium hydroxide (NaOH) and potassium hydroxide (KOH), respectively, were added into the flasks. These mixtures were agitated at 150 rpm for 8 h at 30 °C in shaking incubator (Wise Cube). Afterwards, the adsorbent was removed via vacuum filtration and the quantity of RO16 dye desorbed into the solution was analyzed by spectrophotometry. The desorption efficiency (%) of MA-AC400 was calculated using Equation (20) [36]:

(20) $$\mathrm{Desorption}\;\mathrm{efficiency}=\left(\frac{\mathrm{Amount}\;\mathrm{of}\;\mathrm{RO}16\;\mathrm{desorbed}}{\mathrm{Amount}\;\mathrm{of}\;\mathrm{RO}16\;\mathrm{dye}\;\mathrm{adsorbed}}\right)100$$

## 4. Conclusions

In this study, *Melia azedarach* was used as the precursor biomass for the production of H_3_PO_4_- activated MA-AC400 adsorbent at a comparatively low temperature exposure of 400 °C and contact time of 30 min, biomass and acid ratio 2, compared to the published reports. MA-AC400 was better characterized by SEM, EDS, FTIR, DLS and fluorescence measurements. The fluorescent MA-AC400 is characterized as fine particles with a majority of particles in the range of 0.12 to 0.30 µm and emission fluorescence at 497 nm. The textural properties of MA-AC400 correspond to a mesoporous AC with a BET surface area of 293.13 m^2^ g^−1^ and a Langmuir surface area of 440.83 m^2^ g^−1^, pore volume of 0.149 cm^3^ g^−1^, and average pore width (4V/A by BET) of 20.3 Å. Moreover Boehm titration results confirm the acidic character of MA-AC400 with total acidic functional groups (3.5 mmol g^−1^) and total basic groups of 0.45 mmol g^−1^. The number of phenolic groups was found to be higher, i.e., 1.7 mmol g^−1^, followed by carboxylic acid (0.30 mmol g^−1^) and lactonic (1.5 mmol g^−1^) groups. The chemical interactions between RO16 and MA-AC400 were evidenced from EDS and FTIR analyses. The experimental adsorption capacity of MA-AC400 for RO16 was observed to be 58.54 mg g^−1^ with the adsorbent dosage of 0.4 g L^−1^ and solution pH of 6.2. The RO16 adsorption over MA-AC400 follows PSO and intraparticle diffusion kinetics at lower and higher initial concentrations of dye, respectively. Equilibrium data reveal a better fit to the Langmuir isotherm rather than the Freundlich isotherm with higher R^2^ and maximum Langmuir adsorption capacity predicted as 65.79 mg per gram of MA-AC400. Error analysis confirmed the Langmuir chemisorption for RO16 with higher coefficient of determination and lower values of chi-square error (χ^2^), sum of absolute error (SAE), Marquardt’s percent standard deviation (MPSD) and HYBRID functional error (HYBRID). MA-AC400 adsorbent could be used under suitable conditions to remove anionic RO16 dye and considered to be effectively applied in pilot scale projects. Economic cost of MA-AC400 adsorbent was calculated to be 1388.33 PKR ($8.36 US/kg). It is recommended to explore the potential of this MA-AC400 for the scavenging of toxic metal ions and other emerging contaminants present in industrial wastewater. The testing of this adsorbent in real wastewater treatment technology can further unveil its capabilities and shortcomings. It is further recommended to develop commercial production facility for the development of MA-AC400.

## Figures and Tables

**Table 1:** Textural parameters of MA-AC400 adsorbent.

Parameters	Value
**Surface Area**	
Surface area (S_BET_ m^2^ g^−1^)	293.13
Single point surface area (m^2^ g^−1^)	293.65
Langmuir surface area (m^2^ g^−1^)	440.83
t-plot micropore area (m^2^ g^−1^)	145.65
t-plot external surface area (m^2^ g^−1^)	147.47
BJH adsorption cumulative surface area of pores between 17 Å and 3000 Å width (m^2^ g^−1^)	97.63
**Pore Volume**	
t-plot micropore volume (cm^3^ g^−1^)	0.08
Single point adsorption total pore volume of pores less than 27.190 Å width at p/p_0_ = 0.299 (cm^3^ g^−1^)	0.15
BJH Adsorption cumulative volume of pores between 17 Å and 3000 Å width (cm^3^ g^−1^)	0.05
BJH pore volume (cm^3^ g^−1^)	0.05
**Pore Size**	
Average pore width (4V/A by BET) (Å)	20.33
BJH average pore width (4V/A) (Å)	21.36

**Table 2:** Thermodynamic parameters for RO16 adsorption by MA-AC400.

**ΔH (kJ mol^−1^)**	**ΔS (J mol^−1^ K^−1^)**	**ΔG (kJ mol^−1^)**			
		**T (K)**			
		303.15	313.15	323.15	333.15
−84.79	179.95	−139.23	−141.03	−142.83	−144.63

**Table 3:** Error function analysis for linear isotherm fitness.

Error Functions	Langmuir	Freundlich
R^2^	0.9827	0.9803
χ^2^	0.23	0.61
SAE	4.76	6.71
MPSD	5.58	10.60
HYBRID	11.07	6616.39

**Table 4:** Parameters and constants of PFO, PSO and IPD kinetic models fitness.

Kinetic Model	Parameters	Initial Reactive Orange 16 Dye Concentration (mg L^−1^)				
		20	40	60	80	100
PFO	q_e exp_	14.72	27.73	45.63	41.90	49.23
	q_e cal_	10.12	20.48	34.16	37.49	42.14
	Δq (%)	11.82	9.87	9.51	3.98	5.44
	K_1_	0.02	0.01	0.01	0.01	0.01
	R_2_	0.8530	0.9341	0.9301	0.9446	0.7605
PSO	q_e cal_	16.34	31.95	54.05	58.14	59.17
	Δq (%)	4.16	5.38	6.52	13.71	7.15
	K_2_	0.002	0.001	0.0003	0.0002	0.0002
	R^2^	0.9720	0.9730	0.9185	0.7351	0.7893
IPD	C_i_	8.47	13.68	15.69	8.60	12.43
	K_pi_	0.07	0.16	0.31	0.33	0.36
	R_2_	0.9661	0.9844	0.9409	0.9453	0.8988

**Table 5:** Cost analysis of MA-AC400 production at lab scale (PKR versus USD).

Operations	Activities/Treatment	Cost Breakdown	Cost (PKR)	Cost ($)
Processing of Adsorbent	Collection	Purchased from local market	10.00	0.06
	Grinding	Hours × units × per unit cost = 0.05 × 0.4 × 5.79 *	0.12.00	0.001
	Washing	5 L × 10	50.00	0.30
	Drying	Hours × units × per unit cost = 2 × 0.4 × 5.79	4.63	0.03
Preparation of MA-AC400	*o*-Phosphoric Acid	2 (L) × 300	600.00	3.62
	Nitrogen Gas	1 (Cylinder) × 850	170.00	1.02
	Carbon Dioxide	1 (Cylinder) × 1550	310.00	1.87
	Carbonization	Hours × units × per unit cost = 0.5 × 6 × 5.79	17.37	0.11
	Washing	10 L × 10	100.00	0.60
	Total		1262.12	7.60
	10% (overhead budget)		126.21	0.76
	Total cost		1388.33	8.36

* Unit cost: According to Peshawar Electric Supply Company (PESCO), Pakistan.

**Table 6:** Properties of Reactive Orange 16 dye.

Name	Reactive Orange 16 (RO16)
Synonym	Remazol brilliant orange 3R
CAS Number	12225-83-1
Color Index Number	17757
Empirical formula	C_20_H_17_N_3_Na_2_O_11_S_3_
Molecular weight	617.54
λmax	494 nm
Structure